# The difference is black and green: How functional divergence of an enzyme family gave us diverse teas

**DOI:** 10.1093/plcell/koae272

**Published:** 2024-10-07

**Authors:** Vicky Howe

**Affiliations:** Assistant Features Editor, The Plant Cell, American Society of Plant Biologists; Department of Developmental Genetics, Heinrich-Heine University, Düsseldorf, Germany, 40225

Did you know that, after water, tea is the most widely consumed beverage in the world? In fact, I am sipping a cup of Earl Grey right now. But perhaps you prefer a nice sencha? Aside from being processed differently, black and green teas also differ in the varietals of tea plant (*Camellia sinensis*) used to produce them. So how did we end up with such diverse tea varieties?

Not only has the tea plant been subjected to millions of years of natural selection, but modern cultivars have also undergone extensive artificial selection, resulting in 2 main groups of cultivars that occupy distinct environmental niches. *Camellia sinensis* var. *assamica* (CSA) grows in tropical areas and has relatively large leaves that are not cold tolerant. If left to its own devices, CSA grows as a tree, although cultivated CSA is pruned to facilitate harvesting and is largely used to produce black tea, such as my Early Grey (see Fig). *Camellia sinensis* var. *sinensis* (CSS), on the other hand, grows as a shrub, often in cold, high-elevation environments, and its small, delicately fragrant leaves are generally used to make high-quality green teas.

**Figure.**
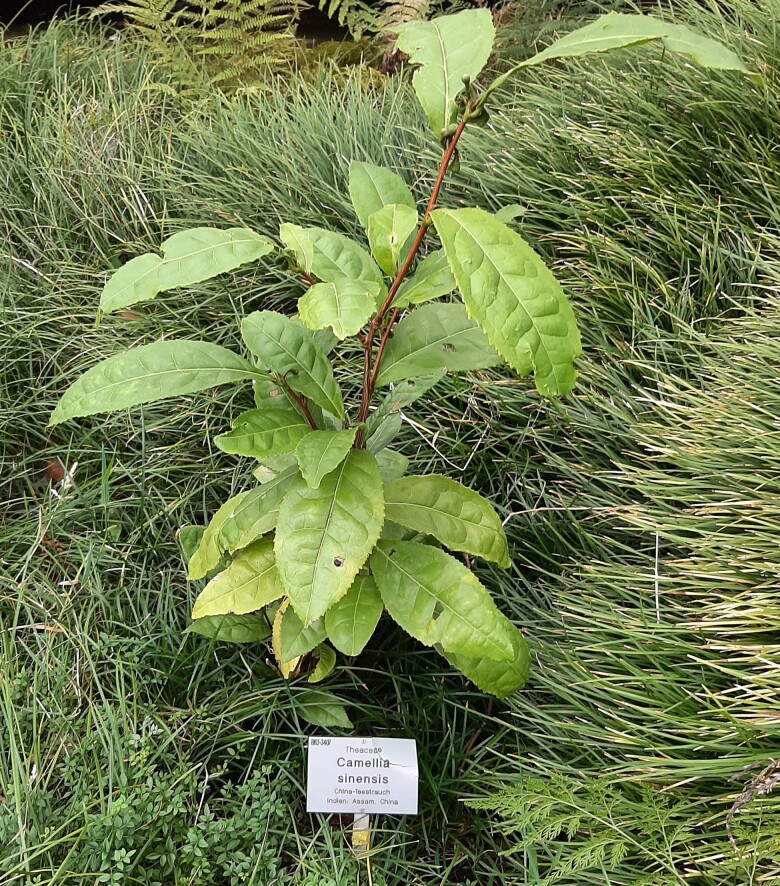
CSA prefers a warmer environment and has large leaves that are generally used to make bold, black teas. This specimen is growing in the glass house at the Botanic Gardens here in Düsseldorf, Germany. Unlike the CSS varietals, it would not survive the frosty winters outside.

As with most crops, humans have been selecting tea plants based on flavor and hardiness for centuries without understanding the genetic and molecular mechanisms underpinning these phenotypic traits. As many of tea's aroma and defense compounds are secondary metabolites that are glycosylated by UDP-dependent glycosyltransferases (UGTs), there could be a link between UGTs and tea quality or stress resilience (Zhang et al. 2024). Genomic studies have revealed that UGTs underwent expansion in the tea plant in recent evolutionary history and that they may have been subjected to artificial selection through the domestication process (Xia et al. 2020). However, until now, functional studies linking the selection of UGTs with differences in tea quality and stress resistance between cultivars have been lacking. To fill this knowledge gap, Wang and coauthors (Wang et al. 2024) examined the evolutionary relationships and looked for functional differences between UGTs in a wild tea plant accession, “DASZ,” for which a genome sequence has been generated (Zhang et al. 2020), CSS, and CSA.

To begin with, they found that both CSS and CSA cultivars had a much higher copy number of a particular class of UGTs designated as the “G group” (∼30 copies) compared with the wild DASZ, which had only 2 copies. This suggests that natural selection and artificial selection in the CSS and CSA cultivars drove the expansion of the G group, which occurred through both whole-genome and tandem gene duplication events. The authors set about investigating whether the duplicated genes had diverged to fulfil different functions, which could account for differences in tea flavor and temperature preference between CSS and CSA cultivars.

First, Wang and colleagues examined the phylogenetic relationship between G group genes in wild tea, CSS, and CSA. They found that CSS cultivars had a distinct branch of G group UGTs that had diverged from CSA and wild tea, and these genes had the hallmarks of artificial selection, such as reduced nucleotide diversity. Upon examination of the substrate preferences of the enzymes encoded by these genes, the authors found that indeed, UGTs from duplicated gene pairs acted on different substrates, producing different flavor compounds. Thus, functional divergence in G group UGTs could potentially account for the differences in tea quality between varieties.

The authors also examined the roles of UGTs in cold and drought tolerance to see if functional divergence could also account for differences in habitat preference between CSS and CSA cultivars. Using RNA silencing to transiently suppress UGTs in different phylogenetic subgroups, they found that the artificially selected UGT genes in the CSS cultivar were involved in cold tolerance but did not respond to drought. This fits with previous studies that suggest genes involved in cold tolerance and flavor metabolism were subjected to stronger selection pressures during the domestication of CSS cultivars than in CSA (Wang et al. 2020). It also implies that the expansion and functional divergence of the UGT G group underlies at least some of the differences in tea flavor and environmental adaptability between the 2 main tea cultivars, although there are likely other factors involved.

Overall, this study reveals that the domestication process has contributed to functional diversification of G group UGTs in tea, accounting for some of the differences in flavor and stress resistance between the 2 main tea cultivars. Given that tea production is highly vulnerable to climate change, knowing which genes are responsible for driving environmental resilience might come in handy if we want to keep enjoying this economically important and delicious drink. Food for thought to go with your next cup of tea!

## Data Availability

No new data were generated or analysed in support of this.

## References

[koae272-B1] Wang J , Hu Y, Guo D, Gao T, Liu T, Jin J, Zhao M, Yu K, Tong W, Ge H, et al Evolutionary amplification and functional divergence of UDP-dependent glycosyltransferases genes shapes the quality traits and cold tolerance of tea plants. Plant Cell. 2024. In press. 10.1093/plcell/koae268 PMC11663605 39365921

[koae272-B2] Wang X , Feng H, Chang Y, Ma C, Wang L, Hao X, Li A, Cheng H, Wang L, Cui P, et al Population sequencing enhances understanding of tea plant evolution. Nat Comm. 2020:11(1):4447. 10.1038/s41467-020-18228-8 PMC7477583 32895382

[koae272-B3] Xia E , Tong W, Hou Y, An Y, Chen L, Wu Q, Liu Y, Yu J, Li F, Li R, et al The reference genome of tea plant and resequencing of 81 diverse accessions provide insights into its genome evolution and adaptation. Mol Plant. 2020:13(7):1013–1026. 10.1016/j.molp.2020.04.010 32353625

[koae272-B4] Zhang J , Yu Y, Qian X, Zhang X, Li X, Sun X. Recent advances in the specialized metabolites mediating resistance to insect pests and pathogens in tea plants (*Camellia sinensis*). Plants. 2024:13(2):323. 10.3390/plants13020323 38276780 PMC10818678

[koae272-B5] Zhang W , Zhang Y, Qiu H, Guo Y, Wan H, Zhang X, Scossa F, Alseekh S, Zhang Q, Wang P, et al Genome assembly of wild tea tree DASZ reveals pedigree and selection history of tea varieties. Nat Comm. 2020:11:3719. 10.1038/s41467-020-17498-6 PMC7381669 32709943

